# Beneficial Hepatokines in MASLD/MASH: Therapeutic Potentials and Drug Delivery Challenges

**DOI:** 10.3390/ijms27167118

**Published:** 2026-08-08

**Authors:** Reeju Amatya, Kyoung Ah Min, Meong Cheol Shin

**Affiliations:** 1 College of Pharmacy and Research Institute of Pharmaceutical Sciences, Gyeongsang National University, 501 Jinju Daero, Jinju-si 52828, Republic of Korea; reejuamatya94@gmail.com; 2 College of Pharmacy and Inje Institute of Pharmaceutical Sciences and Research, Inje University, 197 Injero, Gimhae-si 50834, Republic of Korea

**Keywords:** MASLD, MASH, hepatokines, half-life extension, hepatic fibrosis, protein therapeutics

## Abstract

Metabolic dysfunction-associated steatotic liver disease (MASLD) and its progressive form, metabolic dysfunction-associated steatohepatitis (MASH), are a growing global health burden with extremely limited pharmacological options, thereby indicating an urgent need for innovative therapeutic strategies. Hepatokines are liver-derived secreted proteins that coordinate metabolic communication between the liver and peripheral organs and have emerged as mechanistically compelling therapeutic candidates. This review comprehensively examines beneficial hepatokines with protective roles in MASLD/MASH, detailing their molecular mechanisms of action across key metabolic tissues, including their effects on hepatic lipid metabolism, insulin sensitivity, inflammatory signaling, and fibrogenesis. We further discuss the fundamental pharmacokinetic barriers of these protein therapeutics, particularly their susceptibility to renal clearance and proteolytic degradation. Furthermore, we examine the principal half-life extension strategies to overcome these limitations, with particular emphasis on their clinical application to FGF21-based drug candidates that are currently advancing through phase 3 trials. Overall, this review highlights the therapeutic potential of hepatokine-based biologics and the critical role of protein engineering in translating these mechanistic insights into durable, clinically viable treatments for MASLD/MASH.

## 1. Introduction

Metabolic dysfunction-associated steatotic liver disease (MASLD) has emerged as the most prevalent chronic liver disease worldwide, affecting approximately 25% of the adult population [1,2,3]. MASLD includes a pathological continuum from simple hepatic steatosis through metabolic dysfunction-associated steatohepatitis (MASH), cirrhosis, and hepatocellular carcinoma [4]. Its strong epidemiological linkage to obesity, type 2 diabetes mellitus (T2D), and dyslipidemia suggests the need for therapeutic strategies that address these broader metabolic dysregulations that drive disease initiation and progression [5,6]. The pathogenesis of MASLD is multifactorial and involves hepatic lipid accumulation promoted by increased free fatty acid (FFA) flux from insulin-resistant adipose tissue, enhanced de novo lipogenesis, and impaired mitochondrial β-oxidation [7,8,9]. The downstream consequences thereof, including oxidative stress, endoplasmic reticulum stress, Kupffer cell activation, and hepatic stellate cell activation, mediate fibrogenesis [10]. Despite decades of research, no pharmacological therapy has received approval for the treatment of MASH until very recently [11], thus highlighting the urgent need for innovative therapeutics. In this regard, hepatokines have emerged as an attractive class of drug candidates. These liver-derived secreted proteins function in an autocrine, paracrine, and endocrine manner to coordinate metabolic communication between the liver and peripheral organs [12,13]. The altered secretion of hepatokines reflects and perpetuates the metabolic and inflammatory milieu of liver disease [14,15].

Translating these hepatokines into clinical therapeutics requires overcoming a fundamental pharmacological challenge. Generally, native proteins and peptides exhibit very short plasma half-lives due to renal clearance and proteolytic degradation [16]. However, advances in half-life extension technologies, such as PEGylation, albumin fusion, and Fc-based engineering leveraging neonatal Fc receptor (FcRn)-mediated recycling [17,18], have substantially proven effective in addressing this limitation. Moreover, several strategies have already been applied to hepatokine-based drug candidates with promising results [14,15,19]. This review provides a comprehensive examination of beneficial hepatokines in MASLD, including their molecular mechanisms. We further discuss the pharmacokinetic barriers hindering these protein therapeutics and the engineering strategies being employed to overcome them, with particular focus on their application to hepatokine-based drug development. 

## 2. Beneficial Hepatokines for MASLD/MASH Treatment

**Figure 1:**
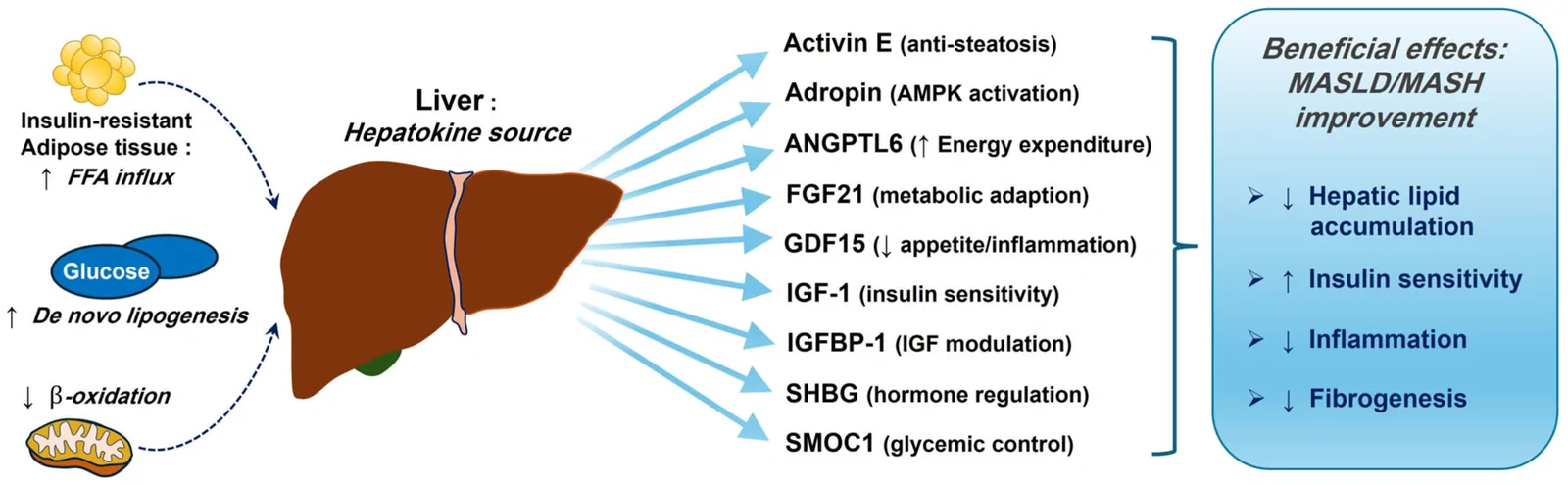
Beneficial hepatokines in MASLD/MASH pathophysiology and therapy. Under metabolic stress conditions, including increased free fatty acid (FFA) influx from insulin-resistant adipose tissue, elevated glucose flux driving de novo lipogenesis, and impaired mitochondrial β-oxidation, the liver responds by upregulating and secreting several beneficial hepatokines. These include activin E (anti-steatotic), adropin (AMPK pathway activation), ANGPTL6 (increased energy expenditure), FGF21 (metabolic adaptation), GDF15 (appetite suppression and anti-inflammatory signaling), IGF-1 (enhanced insulin sensitivity), IGFBP-1 (modulation of IGF bioavailability), SHBG (sex hormone regulation), and SMOC1 (glycemic control). These hepatokines exert autocrine, paracrine, and endocrine effects that counteract hepatic lipid accumulation, improve systemic insulin sensitivity, attenuate inflammatory signaling, and reduce fibrogenic progression, thereby contributing to the improvement of MASLD/MASH pathology. Upward arrow indicates increase, while downward arrow stands for decrease.

The liver serves as an active endocrine hub, secreting various hepatokines that regulate systemic metabolic homeostasis. Hepatokine secretion profiles are substantially altered in MASLD/MASH, reflecting the pathological state of hepatocytes and compensatory adaptive responses aimed at restoring metabolic equilibrium. These liver-derived factors, including activin E, adropin, ANGPTL6, FGF21, GDF15, IGF-1, IGFBP-1, SHBG, and SMOC1, exert pleiotropic effects on metabolic tissues by modulating insulin sensitivity, lipid turnover, energy expenditure, and inflammatory tone. Figure 1 illustrates how metabolic stress in the liver, driven by excess FFA influx, enhanced de novo lipogenesis, and reduced β-oxidation, induces the secretion of beneficial hepatokines and how they improve MASLD/MASH by reducing hepatic lipid accumulation, enhancing insulin sensitivity, and attenuating inflammation and fibrogenesis. Table 1 summarizes the aforementioned nine hepatokines with protective roles in MASLD/MASH.

### 2.1. Activin E

Activin E (encoded by INHBE) is a liver-derived hepatokine that belongs to the transforming growth factor beta (TGF-β) superfamily [20]. The mature secreted form of activin E is a homodimer of approximately ~23 kDa (~14 kDa per monomer subunit) [38]. It promotes energy expenditure by activating brown and beige adipocytes while exerting anti-lipolytic activity in white adipose tissue [38]. The mechanistic basis of its protective function originated from a liver-adipose feedback loop [20]. In response to increased serum free fatty acids (FFAs) and elevated hepatic triglyceride levels, activin E is released from hepatocytes and signals adipose tissue to suppress lipolysis, thereby reducing FFA influx to the liver and preventing excessive hepatic lipid accumulation [20]. At the molecular level, activin E binds directly to activin receptor-like kinase 7 (ALK7), which is primarily expressed in adipose tissue, thereby activating the Smad2 signaling pathway and suppressing the expression of lipolytic genes, including *Hsl* and *Atgl* [21]. Inhbe-knockout mice displayed a lean phenotype but developed severe hepatic steatosis when fed a high-fat diet, as Inhbe deficiency resulted in increased lipolysis in adipose tissue, which resulted in increased fatty acid influx into the liver [20,21]. When the disease phenotype was fully characterized, activin E-KO mice developed pronounced hepatomegaly, hepatic triglyceride accumulation, and histological features consistent with MASH, including steatosis, ballooning degeneration, fibrosis, and increased hepatic crown-like structures. Moreover, the RNA sequencing showed a gene expression characteristic of MASH, including the upregulation of inflammatory and fibrogenic pathways [39].

### 2.2. Adropin

Adropin is a 43-amino acid-long (as the active form) secreted peptide that plays a pivotal regulatory role in energy homeostasis and lipid metabolism [40]. It is primarily expressed in the liver and brain. Adropin promotes AMP-activated protein kinase (AMPK) activation and suppresses SREBP-1c/ACC signaling, thereby improving insulin sensitivity and mitochondrial fatty acid oxidation [40,41]. Furthermore, adropin reduces oxidative stress-induced nuclear factor kappa B (NF-κB) activation via AMPK signaling to attenuate pro-inflammatory cytokine production and Kupffer cell activation [40,41]. Adropin further restrains fibrosis by suppressing inflammatory signaling and inhibiting the activin A/myostatin pathways to reduce fibrogenic gene expression [40,41,42]. In addition, adropin enhances muscle insulin sensitivity and nutrient partitioning via the liver–skeletal muscle axis, thus reducing hepatic triglyceride burden [40,41,42]. Reduced circulating adropin levels have been consistently observed in states of metabolic dysfunction, including obesity, insulin resistance, and MASLD, which suggests that adropin deficiency may contribute to the pathophysiology of these conditions. Moreover, restoration of adropin signaling has been shown to improve hepatic insulin sensitivity, suppress de novo lipogenesis, and enhance mitochondrial oxidative capacity [43,44]. Clinical studies have reported significantly lower circulating adropin concentrations in patients with MASLD compared with healthy controls, with their levels increasing after metabolic interventions, such as dietary modification, exercise, or pharmacological therapy [22,45].

### 2.3. Angiopoietin-like Protein 6 (ANGPTL6)

ANGPTL6, also known as angiopoietin-related growth factor (AGF), is a 470-amino acid-long (~51.7 kDa) hepatokine with metabolically protective properties that primarily functions by stimulating energy expenditure and improving insulin sensitivity. According to Oike et al., more than 80% of AGF-deficient mice died at around embryonic day 13. In contrast, the surviving AGF-deficient mice developed marked obesity, lipid accumulation in skeletal muscle and liver, and insulin resistance with reduced energy expenditure. Furthermore, the hepatic overexpression of AGF via adenoviral transduction (producing approximately a 2.5-fold increase in serum AGF levels) resulted in significant body weight loss and improved insulin sensitivity in mice fed a high-fat diet [46]. Human subjects with higher circulating ANGPTL6 concentrations had a higher resting metabolic rate, and ANGPTL6 significantly improved lipid profiles while also modestly improving insulin concentrations and sensitivity [46,47]. However, despite its beneficial effects on metabolism, human studies have demonstrated a paradoxical increase in ANGPTL6 levels in the serum of patients with metabolic diseases. Qaddoumi investigated the plasma ANGPTL6 levels in patients with obesity and T2D. The ANGPTL6 levels were significantly higher in individuals with obesity and T2D compared with individuals with T2D but not obesity, although individuals with obesity but not T2D had similar ANGPTL6 levels as the controls [48]. A positive association was observed between elevated serum ANGPTL6 levels and fasting blood glucose levels [48]. The difference in circulating ANGPTL6 levels between diabetic and non-diabetic subjects was thought to be due to compensatory mechanisms that counteract hyperglycemia [48].

### 2.4. Fibroblast Growth Factor 21 (FGF21)

FGF21, an 181-amino acid endocrine protein (~19 kDa) predominantly produced by hepatocytes, is the most extensively studied hepatokine for hepatoprotective effects [49,50]. FGF21 has a short plasma half-life due to proteolytic cleavage by dipeptidyl peptidase-4 (DPP4) and fibroblast activation protein (FAP) [51,52]. Hepatic FGF21 expression is strongly induced by fasting, ketogenic states, peroxisome proliferator-activated receptor alpha (PPARα) activation, and metabolic stress, including MASLD/MASH [50,53]. FGF21 may enhance insulin sensitivity, mitochondrial β-oxidation, and lipid turnover by acting via FGFR/β-Klotho signaling [49,54,55]. FGF21 activates AMPK and PPARα, enhancing mitochondrial and peroxisomal β-oxidation, upregulating fatty acid transport and oxidation genes, such as CPT1A, and inhibiting lipogenic enzymes, including acetyl-CoA carboxylase (ACC) and fatty acid synthase (FASN), while suppressing SREBP-1c–driven de novo lipogenesis [49,50,56,57]. Furthermore, FGF21 activates IRS-1/PI3K/AKT signaling in hepatocytes, skeletal muscle, and adipose tissue to promote glucose uptake, glycogen synthesis, and the suppression of gluconeogenesis [50,56,57,58]. FGF21 suppresses hepatic inflammation by inhibiting NF-κB and JNK signaling, thereby reducing pro-inflammatory cytokines, including tumor necrosis factor alpha (TNF-α), interleukin-1 beta (IL-1β), and IL-6, as well as limiting macrophage infiltration [50,56,57]. In addition, FGF21 reduces hepatocyte lipotoxicity and oxidative stress while suppressing TGF-β/SMAD2/3 signaling, which leads to a decrease in collagen I/III and alpha-smooth muscle actin (α-SMA) expression [50,56,57,58]. In the liver–adipose axis, FGF21 induces adiponectin secretion, which feeds back to activate hepatic AMPK and PPARα signaling, thus increasing fatty acid oxidation and indirectly suppressing fibrosis [50,57]. In preclinical studies, FGF21 was shown to reduce hepatic steatosis, inflammatory signaling, and fibrogenesis-associated biomarkers. Elevated FGF21 levels in MASLD have been interpreted as a compensatory response to metabolic stress [50,59]. In a high-fat diet-induced obesity (DIO) model, FGF21 reduced body weight, whole-body fat mass, and blood glucose levels, improved insulin sensitivity, and reversed hepatic steatosis [56]. These findings were consistent with those of clinical studies. In a phase 2a BALANCED trial (NCT03976401), efruxifermin (a long-acting FGF21 analog) produced significant reductions in liver fat by magnetic resonance imaging-derived proton density fat fraction (MRI-PDFF; least squares mean absolute changes from baseline in hepatic fat fraction revealed: −12.3%, −13.4%, and −14.1% in the 28, 50, and 70 mg efruxifermin groups, respectively, vs. 0.3% in the placebo group). In a different phase 2a trial (NCT02413372), pegbelfermin, a PEGylated FGF21 analog, also produced a significant reduction in absolute hepatic fat fraction (10 mg pegbelfermin daily (−6.8%) and 20 mg pegbelfermin weekly (−5.2%) vs. 1.3% in the placebo group). In the phase 2b FALCON 1 trial (NCT03486899) of pegbelfermin, the results demonstrated improved alanine aminotransferase (ALT)/aspartate aminotransferase (AST) levels and fibrogenesis biomarkers (PRO-C3, ELF) and favorably remodeled the lipid profiles with good tolerability [26,60]. Notably, in advanced fibrotic MASH models, FGF21 appears to be capable of decoupling metabolic dysfunction from fibrosis progression, reducing hepatic lipid content, collagen deposition, and profibrotic gene expression [59,61].

### 2.5. Growth Differentiation Factor 15 (GDF15)

GDF15 is a stress-responsive cytokine that belongs to the TGF-β superfamily and is induced in hepatocytes in response to mitochondrial dysfunction, oxidative stress, and inflammatory stimuli [62]. Acting via the GFRAL-RET receptor complex located in the brain, GDF15 potently suppresses appetite, promotes reductions in body weight, and enhances insulin sensitivity, thereby indirectly alleviating hepatic lipid accumulation and steatosis [63,64]. These systemic metabolic effects imply the role of GDF15 as a hepatokine capable of modulating whole-body energy homeostasis in response to liver-derived stress signals. Circulating GDF15 levels are markedly elevated in patients with MASLD/MASH compared to healthy controls and notably correlate with hepatic fibrosis and liver stiffness severity as assessed by histological staging and non-invasive elastography [28,65]. This positive association between GDF15 levels and disease severity appears to conflict with its protective mechanism. However, current evidence supports a compensatory interpretation. Hepatic GDF15 induction is thought to represent an adaptive, stress-induced response to worsening mitochondrial dysfunction, with circulating levels increasing in proportion to hepatocellular injury [66]. This pattern was also reflected in human MASLD cohorts where progressively elevated GDF15 has been interpreted as an active compensatory response to advancing fibrosis risk rather than a marker of inactive disease [67]. This distinction supports the therapeutic rationale in which exogenous, receptor-saturating GDF15 analogs are proposed to restore or amplify a protective signaling axis whose native output, while elevated, may be insufficient to fully counteract progressive hepatocellular stress in instances of advanced disease [68,69].

### 2.6. Insulin-like Growth Factor 1 (IGF-1)

IGF-1 is a 70-amino acid-long protein (mature form) that is primarily produced in the liver via Janus kinase (JAK) 2/signal transducer and activator of transcription (STAT) 5 signaling. Growth hormone (GF) drives hepatic IGF-1 production, and the resulting IGF-1 signal plays a crucial role in postnatal growth as well as energy metabolism [70]. IGF-1 modulates energy homeostasis, inflammatory signaling, and insulin sensitivity [70]. Experimental evidence from liver-specific knockout models of the GH receptor and downstream JAK/STAT signaling components has demonstrated that disruption of this axis led to significant hepatic steatosis, suggesting its indispensable role in maintaining hepatic lipid balance [70]. Clinically, lower serum levels of IGF-1 were significantly associated with worse liver outcomes, including lobular inflammation, hepatocyte ballooning, advanced fibrosis, and MASH [71,72,73,74]. In subjects with histologically proven MASLD, a stepwise reduction in IGF-1 was observed with increasing fibrosis stage, with significantly diminished values at fibrosis stages 3 and 4 [75]. In addition, subjects with steatohepatitis exhibited lower levels of IGF-1 than those with simple steatosis [75]. When cirrhosis develops, a state of hepatic GH resistance ensues, which results in high circulating GH but critically low IGF-1 levels [34]. Clinical trials have demonstrated that GH augmentation reduces hepatic lipid content in individuals with MASLD and may also ameliorate steatohepatitis and fibrosis [34,75,76]. In a 6-month trial of low-dose GH administration in participants with a body mass index (BMI) ≥ 25 kg/m^2^ and MASLD without diabetes, GH administration reduced hepatic steatosis without worsening glycemic measures [77]. Tesamorelin, a GH-releasing hormone (GHRH) analog that augments endogenous GH and downstream IGF-1 secretion, was found to be the only agent to show efficacy in reducing liver fat and preventing fibrosis progression specifically in HIV-associated MASLD in a trial including gold-standard imaging and pre- and post-biopsy sampling [30]. A systematic review and meta-analysis found that GH augmentation significantly reduced alanine aminotransferase (ALT) and gamma-glutamyl transferase (GGT) levels in patients with MASLD [78]. Low-dose recombinant IGF-1 has also demonstrated promising hepatoprotective, antifibrotic, and anti-inflammatory effects, and a small clinical trial in patients with cirrhosis exhibited improved albumin levels and energy metabolism [79,80].

### 2.7. Insulin-like Growth Factor–Binding Protein 1 (IGFBP-1)

IGFBP-1 is a glycoprotein that is predominantly produced by the liver and plays a central role in modulating IGF bioavailability and downstream signaling [81]. Under physiological conditions, the secretion of hepatic IGFBP-1 is tightly regulated at the transcriptional level by insulin, such that circulating levels serve as a sensitive and liver-specific indicator of hepatic insulin action [81,82]. In case of insulin resistance, chronic hyperinsulinemia suppresses IGFBP-1 transcription, thus leading to reduced circulating concentrations [83]. Moreover, fasting serum concentrations of IGFBP-1 are inversely associated with hepatic fat content as measured by proton magnetic resonance spectroscopy [83]. In addition to its utility as a marker of metabolic dysfunction, IGFBP-1 limits the bioavailability of IGF-1. IGF-1 is a known driver of SREBP-1c-mediated lipogenesis via the PI3K/Akt pathway [84]. Therefore, IGFBP-1 would indirectly reduce this pathway by sequestering IGF-1 [85]. Furthermore, the RGD integrin-binding domain of IGFBP-1, through integrin engagement, focal adhesion kinase, and integrin-linked kinase, has been revealed to enhance insulin sensitivity and insulin secretion, with acute administration and chronic infusion both improving glucose clearance and insulin sensitivity in obese mice [86]. In a murine MASLD model established using a methionine- and choline-deficient diet, IGFBP-1 treatment was demonstrated to significantly ameliorate hepatic lipid accumulation by downregulating lipogenesis and upregulating fatty acid β-oxidation, as well as attenuating hepatic inflammation via the inhibition of the NF-κB and ERK signaling pathways [85].

From a clinical perspective, the potential of IGFBP-1 as a non-invasive biomarker has garnered increasing interest. Serum concentrations of IGFBP-1 were positively associated with fibrosis stage in patients with MASLD. Patients with advanced fibrosis (stage 3–4 vs. 0–2) had a significantly higher mean level of IGFBP-1 (29.9 vs. 18.8 µg/L) [87]. This pattern has also been reported across cirrhosis of varying etiologies (viral hepatitis, alcohol-related liver disease, and autoimmune hepatitis), which suggests that it is a general feature of advanced fibrosis rather than specific to MASLD pathophysiology [88]. This reversal likely reflects a shift in the dominant regulatory input on hepatic IGFBP-1 transcription as disease progresses. In early, predominantly metabolic disease, insulin-mediated transcriptional suppression dominates, thereby producing an inverse relationship with hepatic fat and insulin resistance. However, as fibrosis advances, IGFBP-1 transcription becomes increasingly responsive to non-insulin stimuli, particularly the pro-inflammatory cytokines and cellular stress signals that accompany progressive hepatocellular injury, which are known to induce IGFBP-1 expression independent of, and potentially overriding, insulin’s suppressive tone [89,90]. The progressive impairment of hepatocyte insulin signaling within the diseased liver, distinct from whole-body peripheral insulin resistance, may further reduce the capacity of hyperinsulinemia to suppress IGFBP-1 transcription at the hepatocyte level as fibrosis progresses. In this model, IGFBP-1 in early disease functions primarily as a marker of hepatic insulin sensitivity. In contrast, in advanced fibrosis, it predominantly functions as a marker of hepatocellular stress and injury burden, which explains why the two clinical associations point in opposite directions.

### 2.8. Sex Hormone-Binding Globulin (SHBG)

SHBG is a liver-derived glycoprotein that tightly binds approximately 44–65% of circulating testosterone and estradiol, thereby regulating their bioavailability [91]. Hepatic SHBG expression is suppressed by insulin resistance, inflammation, and hepatic lipid accumulation, and it is enhanced by estrogens and HNF-4α activity, thus making circulating SHBG a sensitive marker of hepatic metabolic health [35,92]. Accordingly, SHBG levels are consistently reduced in obesity, type 2 diabetes, and MASLD and inversely associated with steatosis severity [93]. The restoration of SHBG with metabolic improvement correlates with reduced hepatic fat and improved insulin sensitivity [92,94].

### 2.9. SPARC-Related Modular Calcium-Binding Protein 1 (SMOC1)

SMOC1, a mature protein, is a 408 amino acid-long hepatokine. Its gene expression and secretion are upregulated by glucose via a carbohydrate response element-binding protein (ChREBP)-dependent pathway [95]. SMOC1 improves glucose homeostasis by inhibiting cAMP–PKA–CREB signaling in the liver, which leads to decreased gluconeogenic gene expression and hepatic glucose output suppression [37]. Circulating SMOC1 correlated with hepatic and systemic insulin sensitivity and decreased in patients with obesity and insulin resistance. In individuals with insulin resistance and obesity, SMOC1 blood levels were significantly lower than in those without these diseases [37]. The overexpression of SMOC1 in the liver or once-weekly intraperitoneal injections of a stabilized SMOC1-Fc fusion protein induced durable improvements in glucose tolerance and insulin sensitivity in db/db mice, without adverse effects on adiposity, liver histopathology, or inflammation [37].

## 3. Limitations and Technologies to Extend the Plasma Half-Life of Protein Therapeutics

**Figure 2:**
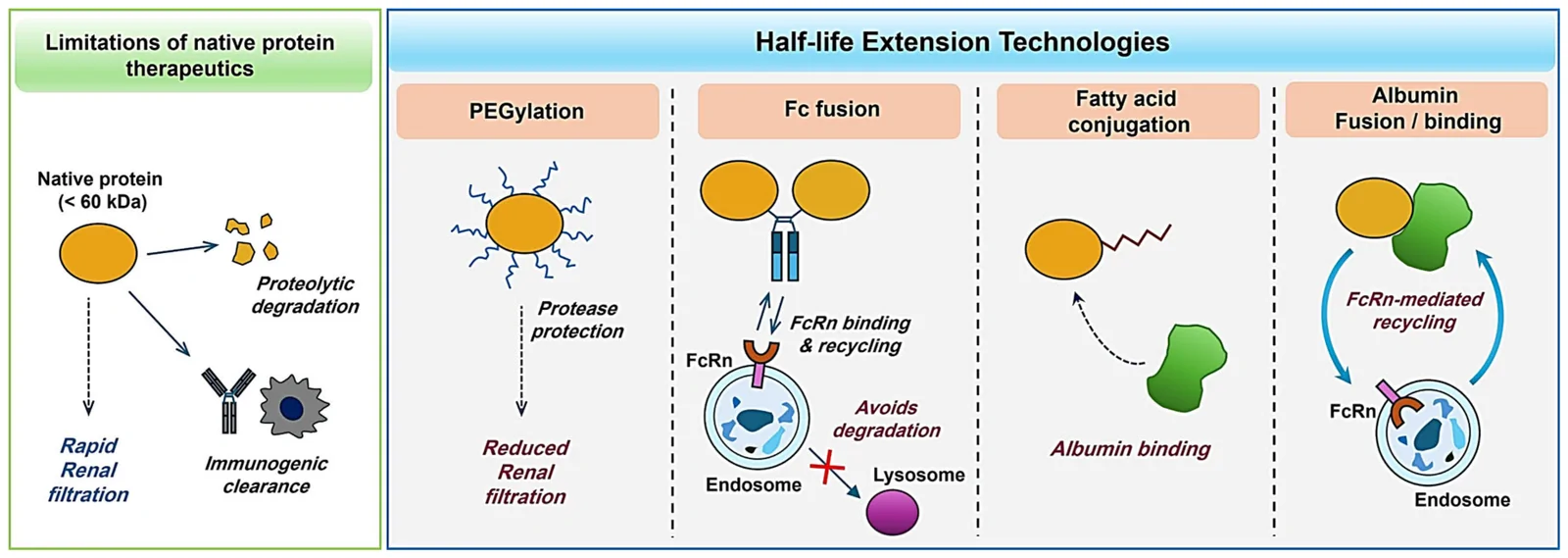
Strategies to extend the plasma half-life of protein therapeutics, illustrated using FGF21 analogs. Native protein therapeutics exhibit short plasma half-lives due to rapid renal filtration, proteolytic degradation, and immunogenic clearance, thus requiring frequent dosing and limiting therapeutic efficacy (left panel). Four principal half-life extension technologies are depicted: (1) PEGylation (e.g., pegbelfermin and pegozafermin), in which the conjugation of polyethylene glycol (PEG) chains increases the hydrodynamic radius and confers protease protection to reduce renal filtration and extend the half-life from hours to ~2 days; (2) Fc fusion (e.g., efruxifermin, efimosfermin, and GDF15/SMOC1 Fc-fusion constructs), which exploits neonatal Fc receptor (FcRn)-mediated binding and endosomal recycling (recycling process indicated by blue arrows) to avoid lysosomal degradation, extending the half-life to days–weeks with weekly to monthly dosing; (3) fatty acid conjugation (e.g., zalfermin), which promotes reversible albumin binding and extends the half-life to approximately one week; and (4) albumin fusion/binding (e.g., human serum albumin (HSA)-FGF21 fusion), which similarly leverages FcRn-mediated recycling within the endosome to achieve half-lives of days to weeks and reduced dosing frequency. Blue arrows in the albumin fusion/binding show FcRn-mediated recycling process.

Protein/peptide-based therapeutics have generated great research interest because of their high specificity and potent biological activity [96,97,98]. Despite these advantages, a major limitation that hinders their clinical translation and long-term efficacy is their intrinsically short plasma half-life [16,99]. The short plasma half-life of protein therapeutics stems from several physiological and biochemical factors. First, the small molecular size renders many peptides susceptible to rapid renal filtration, as proteins below the glomerular filtration threshold (~60 kDa) are swiftly cleared by the kidneys, thus resulting in limited systemic exposure [99,100]. Second, proteolytic degradation by circulating and tissue-resident proteases further reduces their bioavailability [101,102]. In addition, immunogenicity and aggregation may accelerate clearance through uptake by the reticuloendothelial system, thereby further compromising therapeutic durability [103,104]. From a clinical perspective, these limitations translate into the need for frequent dosing regimens that reduce compliance and increase healthcare burden [105]. Moreover, large peak-to-trough fluctuations in plasma drug concentrations may result in suboptimal efficacy or dose-limiting toxicities [16,17]. Overall, extending the plasma half-life has become a critical objective in protein drug development [17]. Figure 2 illustrates the major protein engineering strategies, including PEGylation, Fc fusion, fatty acid conjugation, and albumin fusion/binding, developed to overcome the short plasma half-life of native protein therapeutics caused by renal filtration, proteolytic degradation, and immunogenic clearance, using clinically advanced FGF21 analogs as representative examples in the context of MASLD therapy. Table 2 summarizes the long-acting FGF21 analogs that have been through or are currently in clinical trials.

### 3.1. PEGylation

One of the most widely employed approaches to prolong plasma half-life is chemical modification, particularly polyethylene glycol (PEG) conjugation, known as PEGylation [112]. By covalently attaching PEG chains to proteins, the hydrodynamic radius thereof is increased, reducing renal clearance and shielding the molecules from proteolytic enzymes and opsonin binding [112,113,114]. PEGylation has successfully improved the pharmacokinetics of several approved protein therapeutics and remains a foundation of biologic drug design [113,114].

Pegbelfermin (BMS-986036) is a recombinant human FGF21 analog in which a single PEG chain (~20 kDa) is attached via a site-specific unnatural amino acid (para-azidophenylalanine) [115]. Compared with conventional lysine-based PEGylation, this approach enabled a chemically homogeneous product with the PEG attached at a site that does not interfere with the receptor-binding domains [115,116]. PEGylation increased the molecular weight of FGF21 from ~19 kDa to approximately 30–40 kDa and extended the half-life of the intact C-terminus to approximately 19–24 h, thereby enabling once-daily or once-weekly subcutaneous administration [115,117]. Pegbelfermin also incorporated amino acid substitutions to improve its stability [60,115]. In a phase 2 study (NCT02097277), patients with obesity and T2D received pegbelfermin at doses of 2.5, 10, or 20 mg/day; 5 or 20 mg/week; or a placebo. Daily 20 mg significantly improved high-density lipoprotein (HDL) cholesterol and triglycerides. Liver fibrosis biomarkers (PRO-C3, ELF score) were improved at higher doses, which suggests hepatoprotective activity [60]. In another phase 2a study (NCT02413372), patients with biopsy-confirmed MASH (fibrosis stages F1–F3) received 10 mg/day, 20 mg/week, or placebo. Both active doses significantly reduced the hepatic fat fraction (measured by MRI-PDFF) vs. placebo (−6.8% absolute change vs. −1.3% placebo). Furthermore, the liver fibrosis biomarkers (ELF score, PRO-C3) were significantly improved in the 20 mg/week group [118]. Unfortunately, despite the promising phase 2a trials, two larger phase 2b trials (FALCON 1 in MASH with F2–F3 fibrosis and FALCON 2 in MASH with F4/compensated cirrhosis) did not meet their primary histological endpoints [118,119]. Post hoc analyses suggest that the weekly dosing regimen, which allowed substantial pharmacodynamic recovery between doses due to the relatively short effective half-life, may have been insufficient to sustain the therapeutic benefit required for histological fibrosis improvement [118,119]. In terms of these results, the pegbelfermin program was discontinued.

GlycoPEGylation is a variation in PEGylation in which PEG is attached via a glycan linker, typically exploiting O-glycosylation sites introduced by mutagenesis [120]. Pegozafermin is a glycoPEGylated human FGF21 analog [106]. A 20 kDa linear PEG chain is attached to the protein via an O-glycan at a mutagenesis-introduced site, where a Thr to Ser mutation creates an O-GalNAc acceptor site [121]. In addition to glycoPEGylation, pegozafermin incorporates amino acid substitutions designed to block FAP cleavage at the C-terminus and improve thermostability [121]. The resulting molecule has an in vivo half-life of approximately 50 h in humans, thereby enabling once-weekly or every two-week subcutaneous administration [121]. In the ENLIVEN phase 2b (NCT04929483) study with patients having biopsy-confirmed MASH and F2–F3 fibrosis (with an additional F4 cohort), at 24 weeks, the 44 mg every 2 weeks and 30 mg weekly dose regimens both met the co-primary histological endpoints of fibrosis improvement without MASH worsening (~27% vs. 8% placebo for the F2–F3 cohort), and MASH resolution without fibrosis worsening. In the F4 cohort, 45% of pegozafermin-treated patients achieved ≥1-stage fibrosis improvement vs. placebo. Significant improvements were also observed in liver fat, non-invasive fibrosis markers (ELF score, FIB-4), low-density lipoprotein (LDL), HDL, and triglycerides [106]. In another phase 2 ENTRIGUE (NCT04541186) trial with patients having severe hypertriglyceridemia (triglycerides (TG) ≥ 500 mg/dL and ≤2000 mg/dL), pegozafermin at all tested doses met the primary endpoint of significant triglyceride reduction vs. placebo, with dose-dependent effects across the 9 mg weekly through 36 mg biweekly range [107]. Based on these successful phase 2 trials, the phase 3 ENLIGHTEN-fibrosis trial evaluated pegozafermin in patients with non-cirrhotic MASH and F2–F3 fibrosis, with co-primary endpoints of ≥1-stage fibrosis improvement and MASH resolution at week 52.

Despite these benefits, PEGylation has several well-documented limitations. Pre-existing and treatment-induced anti-PEG antibodies have been detected in patients receiving PEGylated biologics, and these antibodies can induce an “accelerated blood clearance” phenomenon, in which the pharmacokinetic benefit of PEG conjugation is progressively lost with repeated dosing [122]. Because PEG is not biodegradable, high-molecular-weight PEG chains can accumulate intracellularly and produce cytoplasmic vacuolation in the kidney, spleen, and choroid plexus in a molecular-weight-dependent manner. In the liver, PEG immunoreactivity has been observed in Kupffer cells without accompanying vacuolation at the molecular weights tested [123]. Site-specific conjugation, as used for pegbelfermin, mitigates but does not eliminate the risk of PEG interfering with receptor engagement, as the added hydrodynamic bulk may still sterically hinder productive receptor binding even at sites remote from the primary interaction surface. Finally, the outcomes of the pegbelfermin FALCON trial illustrate a more general limitation of PEGylation relative to Fc fusion or fatty acid conjugation: the half-life extension achieved (approximately 19–24 h) may be comparatively modest, and weekly dosing regimens developed on this shorter half-life proved insufficient to sustain the pharmacodynamic exposure required for histological fibrosis improvement [118,119].

### 3.2. Fc Fusion and FcRn-Mediated Recycling

Fusion of therapeutic proteins to the Fc region of IgG exploits the neonatal Fc receptor-mediated recycling to substantially extend plasma half-life [124,125]. Fc fusion increases molecular size, reducing renal clearance, while enabling active recycling from acidic endosomes back into circulation, thereby limiting lysosomal degradation [125,126]. The molecular basis of FcRn-mediated recycling relies on pH-dependent binding, with high-affinity interaction at acidic pH (~6.0–6.5) and release at physiological pH (~7.4) [126,127]. This dual mechanism can extend the half-life from hours to days or weeks and has been broadly applied to various protein therapeutics [125,126,127,128].

Efruxifermin is a bivalent Fc-FGF21 fusion protein. This molecule is a homodimer with a molecular weight of approximately 92 kDa that consists of two identical polypeptide chains, each comprising an IgG1 Fc domain fused at its C-terminus to an FGF21 variant [129,130]. The N-terminal placement of Fc relative to FGF21 was selected to enhance the accessibility of the N-terminal FGFR1c-binding region and C-terminal β-klotho-binding region of FGF21 [129]. The FGF21 moiety of efruxifermin incorporates three key amino acid substitutions: (1) L98R suppresses FGF21 aggregation at high concentrations and elevated temperatures, thus improving formulation stability; (2) P171G prevents FAP-mediated C-terminal proteolytic cleavage, which is the dominant degradation pathway that eliminates β-klotho binding; and (3) A180E enhances binding affinity to mouse, cynomolgus, and human β-klotho, thereby increasing potency and reducing carboxypeptidase-mediated degradation of the C-terminal tail [129]. The IgG1 Fc scaffold mediates FcRn-based recycling. As a result, the N- and C-termini of the FGF21 moiety both have extended and matched half-lives of 3–3.5 days in humans following subcutaneous injection, which enables once-weekly dosing [130,131]. There have been many clinical trials for the efruxifermin. A phase 2a study (NCT01856881) of efruxifermin included patients with T2D. The drug demonstrated significant dose-dependent improvements in insulin sensitivity (HOMA-IR), LDL cholesterol, triglycerides, and HDL cholesterol. The half-life of 3–3.5 days was confirmed, supporting weekly dosing [131]. Another phase 2a BALANCED trial (NCT03976401) included patients with biopsy-confirmed MASH and F1–F3 fibrosis. Treatment with 28 mg or 50 mg of efruxifermin once weekly significantly reduced hepatic fat fraction by MRI-PDFF (−24.7% and −32.9% absolute at highest doses vs. −3.9% placebo) and improved the liver injury markers including ALT, AST, GGT, and adiponectin. Fibrosis biomarkers also exhibited improvement [26]. Furthermore, a phase 2b HARMONY trial (NCT04767529) included patients with biopsy-confirmed MASH and F2–F3 fibrosis. At 96 weeks, 28 mg and 50 mg of efruxifermin weekly demonstrated significant fibrosis improvement. Fibrosis was reduced by ≥39% and 41% vs. 20% for the placebo. MASH resolution was achieved in 47% and 76% of patients in the 28 mg and 50 mg groups, respectively, vs. 15% for the placebo [108,132]. In the phase 2b Symmetry trial (NCT05039450), patients with compensated cirrhosis due to MASH (F4) were included. While the trial did not meet its primary endpoint, long-term data revealed meaningful trends in liver histology and metabolic markers [27]. Currently, three phase 3 trials are underway: (1) SYNCHRONY Histology (NCT06215716), (2) SYNCHRONY Outcomes (NCT06528314), and (3) SYNCHRONY Real-World (NCT06161571).

Efimosfermin is an Fc-fusion protein, with the Fc domain from human IgG1 fused to the N-terminus of the engineered FGF21 variant [109,133]. A key structural distinction of efimosfermin relative to its earlier analogs is its engineered disulfide bond within the FGF21 β-trefoil domain, which stabilizes the three-dimensional structure and further reduces its susceptibility to proteolytic degradation beyond FAP site mutation alone [133,134,135]. The combination of Fc-mediated recycling, structural stabilization through the disulfide bond, and ablation of the FAP/carboxypeptidase cleavage sites [135,136] results in an estimated circulatory half-life of approximately 21 days, thereby enabling once-monthly administration [109,133,137,138]. In a phase 2 study including patients with biopsy-confirmed F2/F3 MASH, participants received 300 mg of efimosfermin subcutaneously once monthly for 24 weeks [139]. The results revealed: (1) 45.2% of efimosfermin-treated patients achieved significant ≥1-stage fibrosis improvement without MASH worsening vs. 20.6% on placebo; (2) a significant 67.7% achieved MASH resolution without fibrosis worsening vs. 29.4% on placebo; and (3) significant liver fat reduction and improvements in glycemic control [139,140].

In addition to FGF21, Fc-fusion analogs of GDF15 and SMOC1 have been developed and investigated in preclinical studies. GDF15 has historically been limited by rapid clearance and physicochemical instability, but it has similarly benefited from Fc-based half-life extension strategies. Fc-fusion engineering, incorporating knob-into-hole technology and glycosylation site mutagenesis, has produced GDF15 constructs with improved solubility, protease resistance, and extended plasma half-life [141]. The daily administration of native SMOC1 protein did not produce lasting effects. In this regard, Montgomery et al. developed an Fc-fusion format to create a stabilized, long-acting version of SMOC1. Once-weekly intraperitoneal injections of the stabilized SMOC1-Fc fusion protein induced durable improvements in glucose tolerance and insulin sensitivity in db/db mice, without adverse effects on adiposity, liver histopathology, or inflammation [37]. Moreover, the administration of the long-lasting SMOC1-Fc fusion protein improved glycemic control, cholesterol, and non-alcoholic fatty liver disease (NAFLD) activity scores in lean and obese mice up to 4 weeks following the final administration of SMOC1-Fc [37].

Fc fusion also carries specific drawbacks. The native Fc domain retains effector functions, including antibody-dependent cellular cytotoxicity and complement-dependent cytotoxicity, which are typically undesired in a metabolic therapeutic; eliminating them requires additional silencing mutations that may compromise Fc stability and increase aggregation propensity [142]. Fc-fusion proteins require mammalian cell expression systems, and the glycosylation patterns at the Fc domain can vary according to the producing cell line and culture conditions, with downstream effects on effector function and potential immunogenicity [125]. The bivalent, homodimeric architecture of standard Fc fusions also roughly doubles the molecular weight of the parent protein relative to that of the unmodified ligand. However, whether this increased size measurably impairs penetration into fibrotic liver tissue has not been directly tested for FGF21-Fc constructs, but it is a reasonable engineering consideration given the general relationship between molecular size and tissue extravasation. Similarly, because FcRn-mediated recycling is a saturable process, very high circulating concentrations of Fc-fusion drugs could in principle outpace available FcRn capacity, though this has not been specifically demonstrated for the FGF21 analogs discussed here.

### 3.3. Fatty Acid Conjugation

Conjugation of fatty acid chains to FGF21 exploits the non-covalent albumin binding to extend circulation half-life [143,144]. Albumin has a half-life of approximately 19–21 days in humans, and fatty acid-conjugated molecules that bind to albumin with appropriate affinity can achieve substantially extended pharmacokinetics [143,144]. Zalfermin employs fatty acid conjugation in combination with multiple stabilizing mutations, thereby yielding a predicted once-weekly dosing profile. The protein scaffold of zalfermin incorporates mutations at key positions to prevent FAP-mediated C-terminal cleavage, reduce aggregation, improve thermal stability, and maintain high-affinity β-klotho binding [110]. Importantly, the fatty acid is conjugated at a site that is remote from the N-terminal FGFR1c-binding region and the C-terminal β-klotho-binding region, thus preserving full receptor activation capacity. In preclinical studies in cynomolgus monkeys, zalfermin demonstrated potent metabolic effects, including reduction in hepatic fat, LDL, and triglycerides with improvements in insulin sensitivity and favorable pharmacokinetics that support once-weekly dosing in humans [110]. In a phase 1 study (NCT03015207) including healthy male participants with a BMI of 25–34.9 kg/m^2^, zalfermin demonstrated dose-dependent pharmacodynamic effects, including reductions in triglycerides and LDL and increases in adiponectin and HDL [111]. Additional phase 1 studies (NCT04722653 and NCT03479892) further characterized the safety, tolerability, and pharmacokinetics of zalfermin across a range of doses [111]. Zalfermin is currently undergoing a phase 2b clinical trial (NCT05016882) to evaluate its efficacy and safety in patients with biopsy-confirmed MASH and liver fibrosis [110].

A key limitation of fatty acid conjugation is that its half-life-extending effect is intrinsically dependent on the concentration and binding capacity of circulating serum albumin, both of which vary among individuals and are frequently reduced in patients with advanced liver disease. In cirrhosis, hepatic albumin synthesis can fall by 60–80% relative to that of a healthy liver, thereby producing hypoalbuminemia that alters the pharmacokinetics of highly protein-bound therapeutics [145]. Moreover, reduced plasma albumin and α1-acid glycoprotein concentrations in severe cirrhosis have been shown to more broadly increase the free fraction of protein-bound drugs [146]. Because MASLD/MASH therapeutic candidates target a population in which a subset will have progressed to or be at risk of decompensated liver disease, this dependency presents the untested possibility that the pharmacokinetic advantage of fatty acid-conjugated agents could be attenuated in patients with more advanced fibrosis. However, this has not, to our knowledge, been directly evaluated for any FGF21 analog and would be a relevant question for future clinical pharmacokinetic studies in this population. Additional limitations include competition with endogenous FFAs and other albumin-bound drugs for the same binding site and the need for careful selection of the conjugation site to avoid interference with the receptor-binding domains, as implemented for zalfermin [110].

### 3.4. Albumin-Based Half-Life Extension Technologies

Albumin-based half-life extension exploits the long circulation time of serum albumin and FcRn-mediated recycling to prolong the systemic exposure of therapeutic proteins, employing strategies such as direct fusion to human serum albumin, genetic fusion to albumin-binding domains, or chemical conjugation to albumin-binding moieties [124,147,148]. In MASLD, albumin-based half-life extension strategies have been successfully applied to beneficial hepatokines. Moreover, the MRI-PDFF-FGF21 fusion protein has been evaluated in high-fat diet mouse models, where the administration of recombinant human serum albumin (HSA)-FGF21 suppressed hepatic lipid accumulation, reduced plasma lipid levels, improved hyperglycemia and hyperinsulinemia, decreased inflammatory cytokines in adipose tissue, and ameliorated liver damage and fibrosis, along with the induction of adiponectin and thermogenic gene expression [149]. These effects are attributable in part to the suppression of hepatic fatty acid uptake (CD36) and lipogenic transcription factors, including SREBP-1c, FAS, SCD-1, and Elovl6 [149].

Furthermore, albumin-based half-life extension strategies share the same hypoalbuminemia-related dependency as fatty acid conjugation, as both strategies rely on adequate circulating albumin for their pharmacokinetic effect and could therefore, in principle, be more variable in patients with decompensated cirrhosis [145,146]. The direct genetic fusion to human serum albumin (~66 kDa) further increases the total molecular size of the therapeutic beyond what Fc fusion alone contributes, which is a consideration for tissue distribution in fibrotic liver, though this has not been specifically characterized for HSA-FGF21. Production of HSA fusion proteins at a clinical scale and preservation of receptor-binding potency despite the fused albumin domain are additional engineering considerations that generally accompany this approach, as reflected in the design constraints described for other albumin fusion and albumin-binding domain therapeutics [147,148,149].

Table 3 summarizes key clinical trials of the long-acting FGF21 analogs discussed in Section 3, including trial phase and primary outcomes, along with their corresponding ClinicalTrials.gov (NCT) identifiers.

### 3.5. Determinants of Translational Success: Target Accessibility, Engineering Adaptability, and Preclinical Validation

Although FGF21, SMOC1, and activin E are each suggested as beneficial hepatokines in MASLD, only FGF21 analogs have advanced to phase 3 clinical development. At the same time, SMOC1 and activin E remain confined to preclinical and early exploratory stages [21,37,150]. This divergence highlights that mechanistic relevance to MASLD pathophysiology is necessary but is not sufficient for clinical translation; target accessibility, amenability to protein engineering, and depth of efficacy validation are equally decisive. Target accessibility is a primary determinant. FGF21 is a small, secreted ligand that signals through a structurally well-characterized receptor complex that comprises FGFR1c and the obligate co-receptor β-klotho, with functionally distinct and independently mappable N-terminal (FGFR1c-binding) and C-terminal (β-klotho-binding) domains [129,130]. This architecture has enabled extensive site-specific modification without compromising receptor engagement, as demonstrated across the engineering strategies discussed in Section 3.1, Section 3.2, Section 3.3 and Section 3.4. SMOC1 is a matricellular glycoprotein that lacks a comparably resolved receptor-level mechanism in metabolic tissue, thus limiting rational engineering [37]. Activin E poses a different accessibility challenge; it specifically signals through activin receptor-like kinase 7 (ALK7) via Smad2/3 phosphorylation [21,38], a single, relatively well-defined receptor axis; however, the therapeutic direction for targeting it remains unresolved. Activin E directly induces Ucp1 and Fgf21 expression in brown adipocytes and, hepatically, suppresses adipose lipolysis to protect against steatosis, thereby positioning it as a protective signal in these contexts [21,38]. Yet a separate activin E resistance hypothesis proposes that in obesity and MASLD, adipose-tissue activin E signaling becomes functionally impaired despite the ligand’s continued secretion, implying that receptor agonism alone may not restore this protective signaling and that the more viable strategy may instead depend on how this resistance arises [150]. Therefore, the engineering adaptability of FGF21 further distinguishes it from the other candidates. As detailed above, the FGF21 scaffold has demonstrated compatibility with PEGylation, glycoPEGylation, Fc fusion, fatty acid conjugation, and albumin fusion, yielding clinically validated half-lives ranging from approximately 19 h to 21 days [60,106,109,110,149]. This versatility reflects the modular domain structure of native FGF21 and the broader precedent established by half-life extension technologies applied to other biologics [112,113,114,124,125]. By comparison, SMOC1 has been subjected to a single engineering approach, Fc fusion, which has only been validated in db/db and diet-induced obese mouse models [37]. Moreover, activin E-directed development remains limited to monoclonal antibody generation and ELISA-based quantification tools without an engineered, half-life-optimized candidate suitable for clinical dosing [150]. Finally, the depth of efficacy validation most evidently differentiates the three candidates. FGF21 analogs have progressed through a complete translational sequence, including preclinical rodent and non-human primate studies; phase 1 pharmacokinetic and pharmacodynamic characterization; phase 2a histological proof-of-concept using MRI-PDFF and biopsy-confirmed fibrosis staging; and phase 2b/3 trials powered for regulatory histological endpoints [26,60,106,108,118,119,130,131,132,139,140]. Notably, the discontinuation of pegbelfermin following the FALCON 1/2 trial was informative rather than disqualifying, indicating that the dosing regimen and sustained pharmacodynamic exposure, rather than the FGF21 mechanism itself, determined the outcome. This lesson was subsequently incorporated into the design of longer-half-life analogs such as efruxifermin and efimosfermin [118,119]. SMOC1 efficacy data remain restricted to murine models [37], while activin E evidence is mechanistically detailed; it is limited to rodent knockout/overexpression models and in vitro brown adipocyte studies, without an investigational new drug (IND)-enabled candidate in active development [21,38,150].

## 4. Conclusions

In conclusion, hepatokines represent a mechanistically diverse class of endogenous therapeutic candidates for MASLD/MASH. The hepatokines reviewed here highlighted distinct nodes in the liver-peripheral organ communication network. Critically, translating these biological insights into durable therapeutics has been dependent on advances in protein engineering, with half-life extension strategies including PEGylation, glycoPEGylation, Fc fusion, fatty acid conjugation, and albumin-based technologies, which have transformed short-lived native proteins into clinically available drug candidates with weekly or monthly dosing profiles. The clinical studies of long-acting FGF21 analogs illustrate how engineering refinements can influence therapeutic outcomes. Integrating a deeper mechanistic understanding of hepatokine biology with increasingly advanced protein engineering platforms will be essential for realizing the full therapeutic potential of this emerging drug class of hepatokines in the treatment of MASLD/MASH.

Two emerging delivery platforms also warrant brief mention. Liver-targeted mRNA-lipid nanoparticle (LNP) delivery is well-suited for hepatokine therapeutics because LNPs already exhibit strong native hepatic tropism via apolipoprotein E-mediated hepatocyte uptake and require no additional targeting modification. Optimized LNP formulations have achieved several-fold improvements in FGF21 expression over earlier platforms, which suggests its potential for transient, endogenous hepatokine expression directly within the liver. This could avoid some of the immunogenicity and repeat-dosing limitations of PEGylated or Fc-fusion proteins, though questions around dosing control and expression duration remain unresolved for metabolic indications. In addition, oral peptide formulation is a second area of interest, motivated by adherence advantages over injectables. Transcellular permeation enhancers, such as SNAC, have enabled clinically meaningful oral bioavailability for semaglutide, despite the absolute bioavailability remaining too low. However, most hepatokine constructs reviewed here are considerably larger Fc- or albumin-fusion molecules rather than small peptides, and enhancer platforms validated at semaglutide’s scale have not been demonstrated for constructs of this size; therefore, oral delivery may be more feasible in the near-term for smaller hepatokine-mimetic peptides than for the larger engineered analogs. Integrating a deeper mechanistic understanding of hepatokine biology with advanced protein engineering and emerging delivery technologies will be essential for realizing the full therapeutic potential of this drug class in MASLD/MASH.

## Figures and Tables

**Table 1:** Beneficial hepatokines for the treatment of MASLD/MASH.

Hepatokine	Level in MASLD	Key Metabolic Actions	Target Organ(s)/Tissue(s)	Key Refs.
Activin E (INHBE)	Induced by lipid stress	Suppresses lipolysis in WAT via ALK7/Smad2; reduces free fatty acid influx; promotes BAT/beige adipocyte energy expenditure	White adipose tissue; brown/beige adipose tissue	[20,21]
Adropin	Reduced in obesity/MASLD	Activates AMPK; suppresses SREBP-1c/ACC; improves insulin sensitivity and mitochondrial FA oxidation; anti-inflammatory via NF-κB; antifibrotic via activin A/myostatin inhibition	Liver (hepatocytes); skeletal muscle	[22,23]
ANGPTL6 (AGF)	Rise in MetS/T2D	Stimulates energy expenditure; improves insulin sensitivity and lipid profiles; KO mice develop obesity, hepatic steatosis, and insulin resistance	Liver; skeletal muscle; adipose tissue	[24,25]
FGF21	Compensatory in MASLD/MASH	Activates AMPK/PPARα; enhances β-oxidation; inhibits lipogenesis (ACC, FASN, SREBP-1c); improves insulin signaling; suppresses NF-κB/JNK inflammation; inhibits TGF-β/SMAD fibrosis	Liver (hepatocytes); white/brown adipose tissue; hepatic stellate cells	[26,27]
GDF15	Correlates with fibrosis severity	Acts via GFRAL–RET in hindbrain; suppresses appetite; reduces body weight; improves insulin sensitivity; indirectly reduces hepatic lipid and fibrosis markers	Hindbrain/area postrema (CNS); liver (indirect, via weight/energy effects)	[28,29]
IGF-1	Stepwise reduction with fibrosis stage	Modulates energy homeostasis, insulin sensitivity, and inflammation; disruption causes hepatic steatosis; low levels correlate with ballooning, fibrosis, and MASH	Liver (autocrine/paracrine); skeletal muscle; systemic/multiple tissues	[30,31]
IGFBP-1	Low in insulin resistance; increased with advanced fibrosis	Regulates IGF-1 bioavailability; limits SREBP-1c lipogenesis; reduces pro-inflammatory cytokines; decreases HSC activation and collagen deposition; suppresses NF-κB/ERK	Liver (hepatocytes; hepatic stellate cells)	[32,33,34]
SHBG	Reduced in obesity, T2D, MASLD	Regulates bioavailability of testosterone and estradiol; marker of hepatic metabolic health; expression suppressed by insulin resistance, inflammation, and hepatic lipid accumulation	Liver (site of synthesis); systemic circulation acting on gonadal/androgen-estrogen target tissues	[35,36]
MOC1	Reduced in obesity/insulin resistance	Upregulated by glucose via ChREBP; inhibits cAMP–PKA–CREB signaling; suppresses hepatic gluconeogenic gene expression and glucose output	Liver (hepatocytes)	[37]

**Table 2:** Engineering strategies and pharmacokinetic properties of long-acting FGF21 analogs in clinical development for MASH.

Agent	Engineering Strategy	Half-Life (Human)	Dosing Frequency	Molecular Weight	Status	Reference
Pegbelfermin	PEGylation (site-specific)	19–24 h	Daily or weekly (SC)	~30–40 kDa	Discontinued (2021)	[60,106]
Pegozafermin	GlycoPEGylation	~50 h	Weekly or biweekly (SC)	~30 kDa	Phase 3 (MASH, SHTG)	[106,107]
Efruxifermin	Bivalent IgG1 Fc-FGF21 fusion (homodimer)	3–3.5 days	Once weekly (SC)	~92 kDa	Phase 3 (MASH)	[58,108]
Efimosfermin	Disulfide-stabilized FGF21 + Fc fusion	~21 days (estimated)	Once monthly (SC)	~75 kDa	Phase 3-ready (MASH)	[109]
Zalfermin	Protease-resistant mutation + fatty-acid conjugation	~7 days (predicted)	Once weekly (SC)	~23 kDa	Phase 2b (MASH)	[110,111]

**Table 3:** Summary of clinical trials of long-acting FGF21 analogs.

Agent	NCT Number	Trial Name/Phase	Key Outcome	Status
Pegbelfermin	NCT02097277	Phase 2	Daily 20 mg improved HDL-C, triglycerides; fibrosis biomarkers (PRO-C3, ELF) improved at higher doses	Completed
Pegbelfermin	NCT02413372	Phase 2a	Hepatic fat reduction (MRI-PDFF −6.8% vs. −1.3% placebo); ELF, PRO-C3 improved (20 mg/week)	Completed
Pegbelfermin	NCT03486899	FALCON 1, Phase 2b	Did not meet primary histological endpoint (24–31% vs. 14% placebo, *p* = 0.134)	Discontinued (2021)
Pegbelfermin	NCT03486912	FALCON 2, Phase 2b	Did not meet primary histological endpoint	Discontinued (2021)
Pegozafermin	NCT04929483	ENLIVEN, Phase 2b	Co-primary endpoints met (~27% vs. 8% placebo, F2–F3); 45% ≥1-stage improvement in F4 cohort	Completed
Pegozafermin	NCT04541186	ENTRIGUE, Phase 2	Significant, dose-dependent TG reduction vs. placebo	Completed
Pegozafermin	NCT06318169	ENLIGHTEN-Fibrosis, Phase 3	Co-primary: ≥1-stage fibrosis improvement + MASH resolution at wk 52; primary completion est. 2029-02	Recruiting
Efruxifermin	NCT01856881	Phase 2a	Improved HOMA-IR, LDL-C, TG, HDL-C	Completed
Efruxifermin	NCT03976401	BALANCED, Phase 2a	Hepatic fat reduction (−24.7%, −32.9% vs. −3.9% placebo)	Completed
Efruxifermin	NCT04767529	HARMONY, Phase 2b	Wk 96: ≥1-stage fibrosis improvement in 46% (28 mg)/75% (50 mg) vs. 24% placebo (biopsy subset)	Completed
Efruxifermin	NCT05039450	SYMMETRY, Phase 2b	Missed primary endpoint at wk 36; encouraging trends through wk 96	Completed
Efruxifermin	NCT06215716	SYNCHRONY Histology, Phase 3	Ongoing	Recruiting
Efruxifermin	NCT06528314	SYNCHRONY Outcomes, Phase 3	Ongoing	Recruiting
Efruxifermin	NCT06161571	SYNCHRONY Real-World, Phase 3	Ongoing	Recruiting
Efimosfermin	NCT04880031	Phase 2	45% vs. 21% ≥1-stage fibrosis improvement (*p* = 0.038); 68% vs. 29% MASH resolution (*p* = 0.002)	Completed
Efimosfermin	NCT07221227	ZENITH-1, Phase 3	Ongoing	Recruiting
Efimosfermin	NCT07221188	ZENITH-2, Phase 3	Ongoing	Recruiting
Zalfermin	NCT03015207	Phase 1	Dose-dependent TG/LDL-C reduction; increased levels of adiponectin and HDL-C	Completed
Zalfermin	NCT04722653	Phase 1	Safety, tolerability, PK	Completed
Zalfermin	NCT03479892	Phase 1	Safety, tolerability, PK	Completed
Zalfermin	NCT05016882	Phase 2b	Efficacy/safety evaluation ongoing	Ongoing

## Data Availability

No new data were created or analyzed in this study. Data sharing is not applicable to this article.
